# Evaluation of a Health Communication Campaign to Improve Mosquito Awareness and Prevention Practices in Western Australia

**DOI:** 10.3389/fpubh.2019.00054

**Published:** 2019-03-19

**Authors:** Abbey Potter, Andrew Jardine, Annette Morrissey, Michael D. A. Lindsay

**Affiliations:** ^1^Medical Entomology, Environmental Health Directorate, Public and Aboriginal Health Division, Department of Health, Perth, WA, Australia; ^2^Communications Directorate, Office of the Director General, Department of Health, Perth, WA, Australia

**Keywords:** communication campaign, KAP survey, mosquito-borne disease, mosquito(es), public health

## Abstract

*Fight the Bite* represents the Department of Health's first attempt to actively raise awareness and improve prevention practices related to mosquitoes in Western Australia (WA). The multi-faceted campaign model involved a range of stakeholders and delivery methods over a 2 year period, achieving a recall rate of 8.2% among 2,500 survey participants. Significant regional differences were noted in campaign exposure, reflecting the variation in mosquito management issues throughout the State, and subsequent engagement by local government. Of those individuals with campaign recall, 43.8% reported an increase in awareness and 27.4% reported a change in behavior, which equated to a 1.7 and 1.2% change across the total survey population, respectively. The results of this study demonstrate that *Fight the Bite* has significantly improved awareness and prevention practices among those individuals who were exposed to the campaign. This was particularly promising, given the modest budget, resources, and time period over which the campaign was run prior to evaluation. This outcome means that *Fight the Bite* can be confidently adopted as a proven and standardized but regionally adaptable campaign approach to raising awareness about mosquito avoidance and mosquito-borne diseases by the Department of Health and its stakeholders. Future campaign aims include increasing reach through heightened and sustained promotion of *Fight the Bite* by both the Department and local government, as well as expanded collaboration with a range of stakeholders within the community.

## Introduction

Globally, mosquito-borne diseases pose a major threat to public health (1). In Australia, local mosquitoes can transmit Ross River virus (RRV), Barmah Forest virus (BFV), West Nile virus (Kunjin substrain) (WNV_KUN_), and the potentially fatal Murray Valley encephalitis (MVE) virus (2). Whilst there are currently no vaccines or specific treatments for the clinical diseases associated with these infections, they are all preventable through practicing effective mosquito avoidance. It is therefore critical that mosquito management programs engage a range of stakeholders and incorporate a variety of delivery methods in their education efforts to raise awareness, motivate behavioral changes, and in turn reduce the incidence of disease (3).

In the past two decades, there has been significant global growth in our knowledge of interventions required to raise awareness and change health behaviors related to specific aspects of mosquito management. This has largely been limited to the use of community-based interventions aimed at controlling populations of container-breeding vector species through source reduction, and reducing the incidence of mosquito-borne diseases such as dengue and malaria through insecticide treated bed net usage (4–10). Yet there remains a general paucity of data in relation to the importance of incorporating an all-encompassing communication strategy into mosquito management programs.

Historically, little emphasis has been placed on the need for active mosquito-related education in Australia as key vector species, *Aedes albopictus* and *Aedes aegypti*, and their associated viruses are absent (with the exception of *Ae. aegypti* and dengue in northern Queensland), perhaps resulting in a decreased perceived need for a coordinated communication strategy. This has certainly been the case in Western Australia (WA), where the Department of Health (the Department) has largely relied on the use of media statements in response to viral detections, extreme weather events or increased mosquito abundance, website information, and passive distribution of brochures only. Yet over the past decade, health authorities across the country have begun to recognize the importance of integrated mosquito management and the complementary role that education plays, alongside more traditional methods of mosquito control, in reducing the incidence of preventable diseases. In response, a number of communication campaigns have emerged including *Eliminate Dengue* in northern Queensland, *Fight the Bite* in South Australia, and *Beat the Bite* in Victoria.

Following a baseline survey that highlighted gaps in the knowledge and prevention practices of individuals throughout WA in relation to mosquitoes and mosquito-borne disease, the Department recognized the need to overhaul its communication strategy and launched *Fight the Bite* (*FTB*). The multi-faceted, sustainable campaign model involved the Department managing consistency in messaging/artwork, fostering stakeholder relationships and developing a suite of *FTB* resources, whilst more than 35 local government stakeholders were responsible for implementing initiatives at the community level. This combined approach resulted in community-based programs being reinforced by a modest amount of media coverage in all high-risk regions of the state. The significant uptake by local government was attributed to the early and ongoing engagement between the Department and local government, ensuring resources and campaign initiatives were tailored to the needs of their individual mosquito management programs. The broader campaign success was attributed to the successful collaboration with a range of stakeholders including a number of teams within the Department (Environmental Health, Communications, and WA Country Health Service), local governments, the WA branch of the Australian Medical Association, commercial outdoor cinemas, schools, outdoor recreational groups, and employers in mining/resources sector. This paper reports the results of an evaluation survey undertaken after a 2 year pilot period, to determine the efficiency and effectiveness of the campaign model and the impact it has had on public awareness and prevention practices in relation to mosquitoes in WA.

## Materials and Methods

### Baseline Survey

In 2014, the Department conducted an extensive baseline survey throughout the State to ascertain the knowledge, attitudes, and practices (KAP) of individuals in relation to mosquitoes and mosquito-borne disease (11). The survey instrument consisted of 34 questions that required a mix of both open and closed questions.

The results of the survey (published elsewhere) highlighted the need for the Department to overhaul its existing communication strategy, which then became the impetus behind the roll out of *FTB* in WA (11). It also served as a baseline against which to compare any future campaign evaluation results.

### Campaign Background

*Fight the Bite* represents the Department's first attempt to actively raise mosquito awareness and improve prevention practices in WA. The campaign calls for people to *Cover Up. Repel. Clean Up*, and encourages the use of long, light-colored, loose-fitting clothing, application of an effective mosquito repellent and disposal, emptying or covering of water-holding containers where mosquitoes may breed. While *FTB* was originally developed by the Government of South Australia (SA Health), permission was given to the Department to roll the campaign out in WA, tailor existing resources and develop a range of new initiatives that would meet the state's unique mosquito management requirements. The Department partnered with a number of local governments (collectively formed the intervention regions), who regularly report a significant mosquito problem and/or mosquito-borne disease risk, to develop campaign materials and targeted initiatives. The environmental health officers within each local government were actively encouraged to promote the campaign during the 2 year evaluation period and were provided the opportunity to apply for Departmental funding to assist in their efforts. The remaining local government jurisdictions throughout the State, who were not actively involved in this campaign for the entire duration of the evaluation period, formed the control regions.

### Campaign Content

The overall campaign objectives were to increase awareness of the health risks associated with mosquitoes, improve prevention practices, and in turn reduce the incidence of both locally acquired and exotic mosquito-borne diseases among WA residents. In order to achieve this, the Department expanded on SA Health's resources to create a toolkit of visually effective communication materials for stakeholders that targeted individuals at home, on holiday in Australia and on holiday overseas.

Whilst traditional resources included brochures and posters, a suite of digital infographics and suggested posts were developed for use on Facebook and Twitter. Media releases were used to generate unpaid media opportunities via both radio, television, and print. In various regions, depending on local government and Departmental budgets, a small amount of paid advertising was also undertaken. Examples included regional newspapers, geotargeted Facebook advertising, a billboard, train station shopalites, tourist maps, radio, television, outdoor cinema advertising, and the presence at the international airport departure lounge. Environmental health officers worked in partnership with their marketing teams to set up stalls at community events to increase public awareness more actively. A direct mailout was undertaken by the Department to targeted Public Health Units and general practitioners to encourage medical centers to promote the campaign. The WA branch of the Australian Medical Association assisted the Department in their efforts by publishing a journal article and several articles in their online newsletter targeting doctors, during the peak mosquito season. Digital material was also provided to a third party associated with medical clinic advertising, who agreed to air campaign material on a small number of waiting room screens at no cost.

Targeted materials were also developed for specific recreational groups identified by the baseline survey as being at higher risk of mosquito exposure (11). In some regions, members of sporting/recreational groups (e.g., fishing, caravan, and camping clubs) were provided with a *FTB* branded water-proof bag that contained repellent and information on how to prevent being bitten. Campaign branded stickers were placed on bait freezers at local retailers to provide individuals with a timely reminder of the need to protect themselves. A visually effective, counter-top display box was designed to house brochures and a bulk pack of repellent for communal use at a range of locations/establishments including outdoor restaurants, sporting facilities, and mine sites. Community and outdoor cinema stakeholders were provided with a similar kit that included a screen advertisement to increase repellent uptake prior to the movie screening. A number of cinemas opted to screen advertising for free, as they felt they had a duty of care to their patrons.

### Study Area

**Figure 1:**
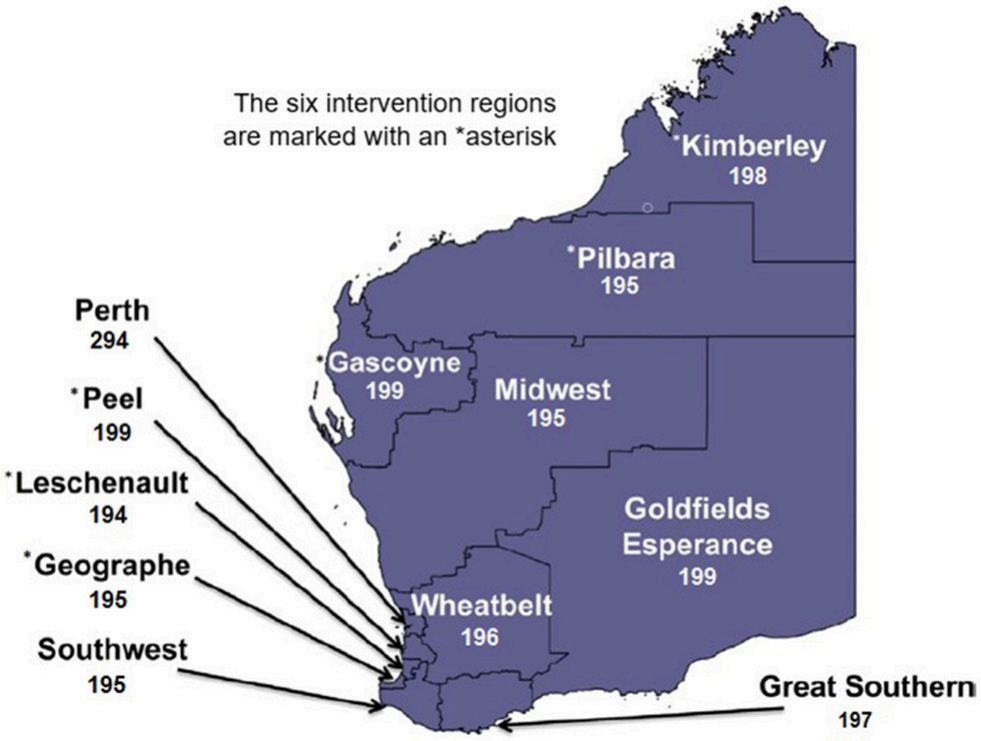
The number of survey respondents from each region in Western Australia (total: 2,456).

WA occupies the entire western third of Australia, with a total land area of 25,269,875 km^2^ (Geoscience Australia, 2001). The majority of the State's population resides in the capital city of Perth, and towns within the southwest corner of WA (12). In this study, a total of 12 geographical regions were surveyed (Figure 1). *FTB* was intensively promoted by local governments within the northern regions of the Kimberley, Pilbara, and Gascoyne, as well as the southwest regions of Geographe, Leschenault, and Peel (Figure 1). These formed the intervention regions. The Goldfields-Esperance, Great Southern, Midwest, Southwest (other) and Wheatbelt regions were not actively involved in the promotion of *FTB* for the entire duration of the 2 year pilot period and served as controls. It is important to note that whilst the authors largely consider Perth to be a control, a minor amount of *FTB* promotion took place in the metropolitan region during the evaluation period. This was because a small number of local governments located within Perth actively wanted to be involved in the campaign during the pilot period (8 local governments), given mosquito breeding and/or increased disease risk is a significant issue for them. The majority of local governments within the jurisdiction were not involved in the campaign (22 local governments).

### Evaluation Survey

A repeat survey of 2,500 individuals, comparable to the baseline study described by Potter et al. (11), was conducted between February and May 2017 to recapture representative KAP data and evaluate *FTB*. The computer assisted telephone interview (CATI) survey was developed by the Department's Medical Entomology team. The Survey Research Center (SRC) at Edith Cowan University was paid to formally conduct the survey (no affiliation with the authors). The SRC are permitted to conduct low risk, commercial surveys with respondents over the age of 18 years without specific ethics approval if the study is a general population survey, the topic is not sensitive, no individual identifying information is supplied, and the samples are drawn from the electronic white pages, determined by the ECU Human Research Ethics Committee (HREC) and Association of Market, Opinion and Social Research Organization (AMSRO). This study fulfilled all of the aforementioned conditions. Furthermore, the study was also captured under the multicenter research project ethics approval granted to the WA Department of Health by the ECU HREC for provision of survey data collection services (Ref: 10185).

While the majority of questions from the baseline survey were repeated in this study, additional questions were added to allow for campaign evaluation. To ensure the survey remained at an optimal time of 12 min, a small number of questions that did not yield significant or meaningful results in the baseline survey were also removed. One noteworthy change was made to the potential list of answers that corresponded to the question “*from which sources have you obtained information regarding mosquitoes and mosquito-borne disease?*.” This remained a closed question for which a list of options were read out that required a *yes* or *no* answer, but due to the frequency in which “*radio”* and “*work”* were mentioned in the “*other (please specify)*” option in the baseline survey, these were included as specific options that were read out to the respondent.

The number of respondents from each of the 12 geographical regions throughout the State is detailed in Supplementary Table 1. Given the highly uneven population distribution in WA, with over three quarters of the state's population located within the Perth metropolitan region (12), all regions except Perth were over-sampled in order to obtain reliable estimates. Households were selected at random by postcode using the electronic white pages listing landline telephone numbers in WA from a pool of 103,354 households. As this is a similar pool used by (11), it is possible (albeit unknown) that subjects included in the baseline study were resurveyed in the evaluation. The surveyor first requested to speak to a male in the household with the next birthday in order to reduce the over-sampling of women noted in the baseline survey (11).

In some instances, respondents replied to questions with a descriptive answer that the surveyor recorded in an “*other”* or “*additional information”* option. Following the completion of the survey, each descriptive answer was assessed and where appropriate, the response was recorded against a pre-coded option if one existed. This was particularly important when individuals were asked whether they had “*heard or seen any specific advertising or educational campaigns related to mosquitoes or mosquito-borne disease in the past 2 years*.” If they answered “*yes,”* they were then asked to recall as much as information as possible. In instances where the *FTB* slogan, calls to action or a specific campaign initiative was described, the response was recorded as an “*unprompted recollection of FTB*.” Individuals who described mosquito-related communication that was not related to *FTB* or provided insufficient information to determine whether it was campaign-related, were recorded as having “*unprompted recollection of other material.”* If a survey respondent had no recollection of any mosquito-related communication, the interviewer provided a final prompt question to confirm that they hadn't seen or heard anything related to *FTB*. The answers were then recorded as either “*no recollection of FTB”* or “*prompted recollection of FTB*.”

### Data Analysis

**Figure 2:**
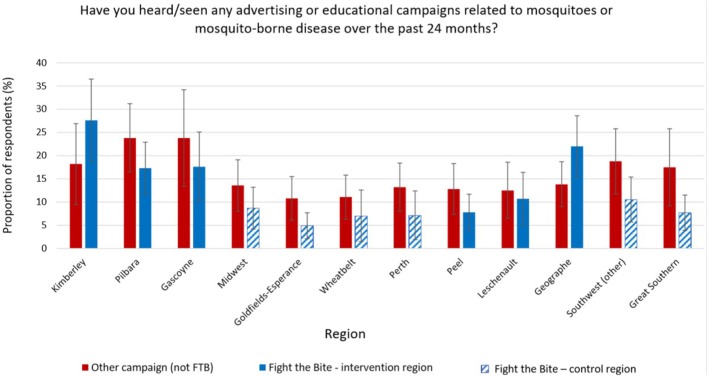
Recall of *Fight the Bite* and other mosquito-related material across the 12 regions surveyed in Western Australia (with 95% confidence intervals).

**Figure 3:**
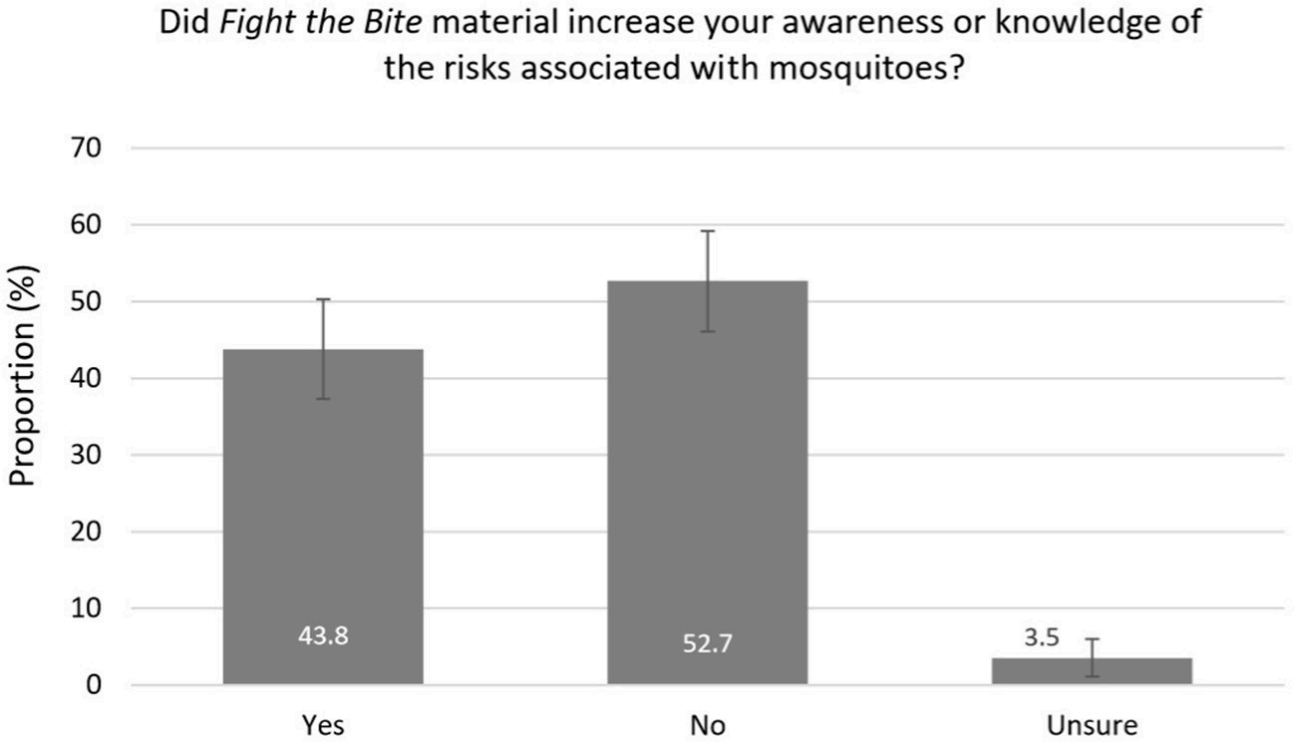
Impact of *Fight the Bite* on mosquito awareness among survey respondents with campaign recall (with 95% confidence intervals).

**Figure 4:**
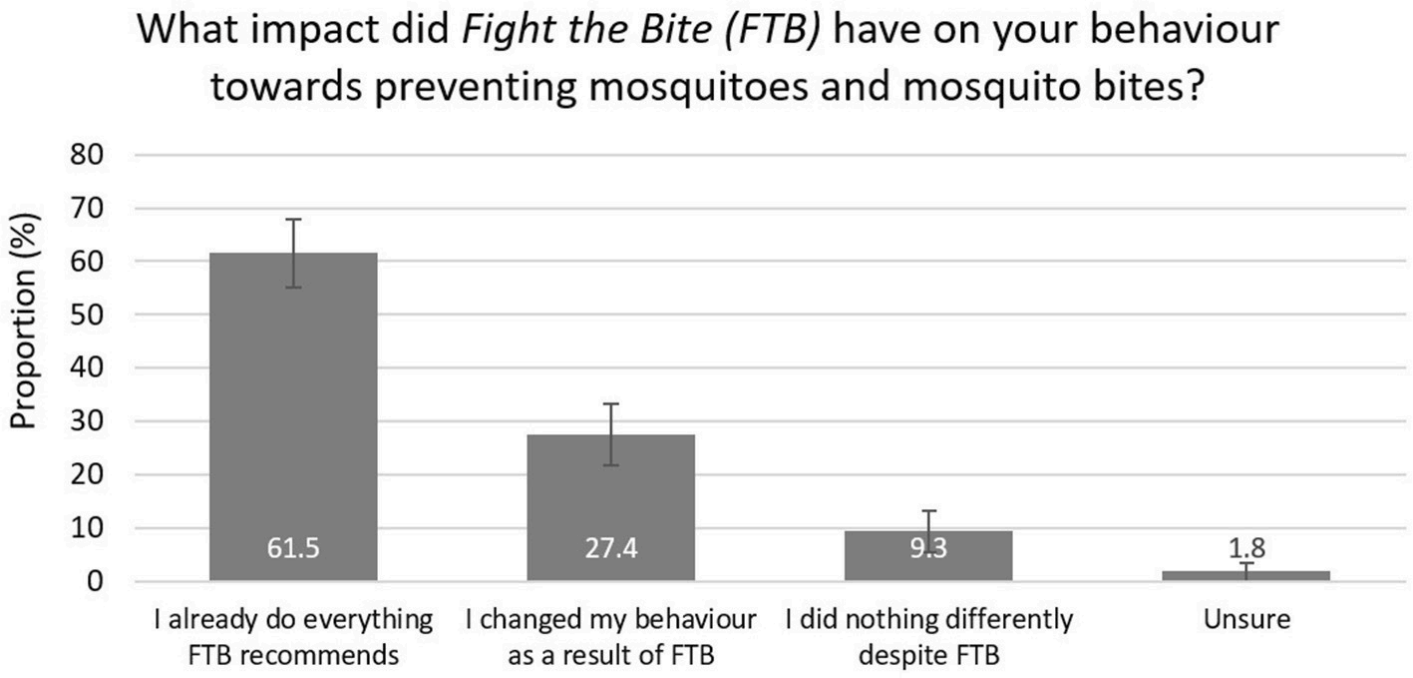
Impact of *Fight the Bite* on behavior and prevention practices among survey respondents with campaign recall (with 95% confidence intervals).

**Figure 5:**
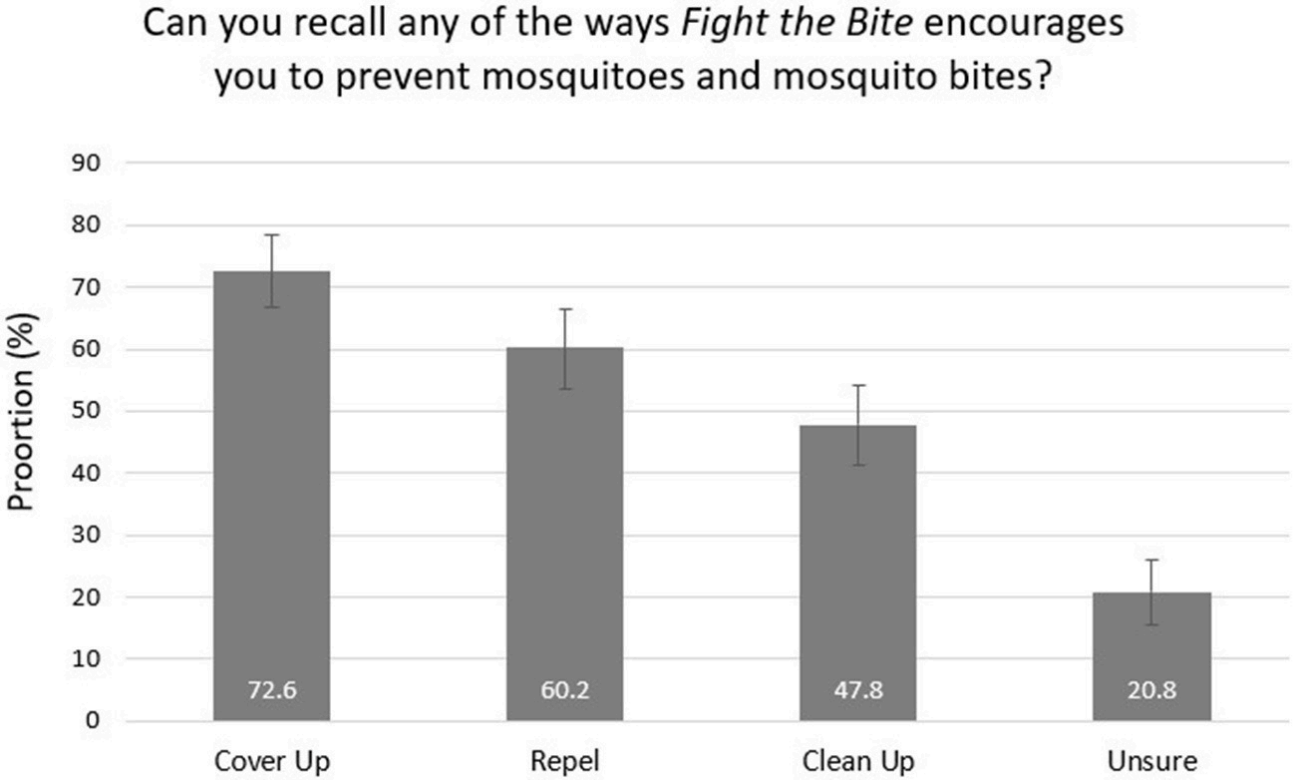
Recollection of key health messages among survey respondents with campaign recall (with 95% confidence intervals).

**Figure 6:**
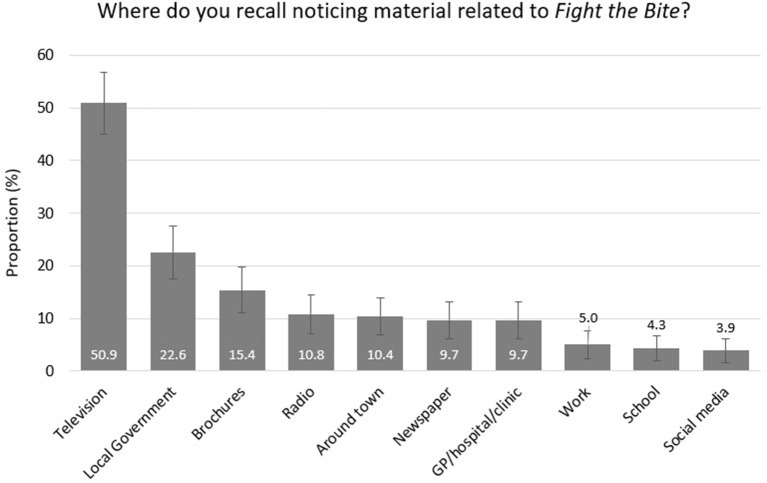
Advertising and promotional mediums noticed by survey respondents with *Fight the Bite* recall (with 95% confidence intervals).

In order to generate representative estimates and facilitate comparisons between subgroups, the survey data presented in Supplementary Tables 2–4 were weighted by age, sex and region to the 2016 estimated resident population (12). The weighting variable was calculated for each survey respondent using age, sex, and region as auxiliary variables and applied when calculating the survey proportions using STATA 11 to ensure the results were representative (StataCorp LLC, College Station, Texas 2011). Regions in Supplementary Tables 2–4 are presented from north to south to make it simpler to visualize and compare regional differences. The data presented in Figures 2–6 represents the subset (*n* = *226*) of the survey population who were able to recall *FTB* and reported changes in awareness and prevention practices following campaign exposure, for which an unweighted analysis was undertaken. This subset of the data was not broken down by region, age or gender as the sample size was too small.

A minimum sample size of 200 for each region was required, to be sufficiently representative and to provide an estimate for each region with a relative standard error of +/– 7% at a confidence level of 95%. For those results that appear in text only (not in table form), the 95% confidence intervals are reported in parentheses. Differences were defined as statistically significant if the 95% confidence intervals of two estimates did not overlap. This is a conservative test of significance that reduces the significance level (alpha) for each individual comparison (13). Reducing the alpha level is appropriate when undertaking multiple comparisons, as is the case in this paper, to minimize the risk of reporting false significant differences due to chance (type 1 error) (14). As such, exact *p*-values were not calculated. Where confidence intervals do not cross over, the difference is always significant, representing a *p*-value < 0.05. However, when there is an overlap by less than approximately one quarter, the *p*-value may still be <0.05. Due to this conservative measure, a number of findings that tended toward significance (i.e., overlap by less than one quarter) have been highlighted in this study. All other differences discussed in the results section of this paper were considered significant by this measure.

## Results

Of the 3,425 households contacted by the SRC during the evaluation survey, 2,511 individuals (one per household) agreed to participate, whilst 914 declined. This resulted in an overall participation rate of 73.3%. As age was one of the variables used to weight the survey data, the 55 respondents who did not provide their age were excluded from the analysis, reducing the total sample size included in the statistical analysis to 2,456. The individuals excluded from the analysis on the basis that they did not give their age were spread evenly across all regions (Supplementary Table 1). Of this final survey sample, 1,252 individuals were male and 1,204 were female. Younger individuals, particularly in the 18–34 years age group, were under-represented in this study as they were less likely to use a registered landline than older age groups (Supplementary Table 1).

### Knowledge

Overall, 63.9% of respondents knew Ross River virus (RRV) was a locally acquired mosquito-borne disease but knowledge of other mosquito-borne diseases in WA was poor (Supplementary Table 2). While there was no significant difference in general knowledge of mosquito-borne diseases between intervention and control groups, awareness of RRV was higher than the State average among individuals from the southwest (Leschenault, Geographe, and Other), Gascoyne and Midwest. Awareness of Murray Valley encephalitis (MVE) was higher than the State average among respondents from the northern regions of WA (Kimberley, Pilbara, and Gascoyne). Despite the tendency for knowledge of RRV and MVE to improve with age, it was interesting to note awareness of Barmah Forest virus was highest among those aged 18–34 years. Fatigue (66.6%) was the most commonly recognized symptom of RRV, followed by painful/swollen joints (46.6%), fever (38.4%), and painful/aching muscles (37.3%). Skin rash (8.8%) was identified by a significantly smaller proportion of respondents. Less common symptoms associated with RRV, including headache, dizziness, sore throat, swollen lymph nodes, loss of concentration and tingling in the hands and feet, were also listed by a small proportion of respondents.

General information regarding mosquitoes and mosquito-borne disease was most commonly sourced from family and friends, television, and traditional print media (Supplementary Table 2). Regional and age differences existed in where individuals most commonly sourced information on this issue (Supplementary Table 2). The influence of local government was more prominent in the northern intervention regions (Kimberley, Pilbara, and Gascoyne) compared to all controls, with the exception of the Wheatbelt (small overlap in confidence intervals). Local government input was significantly greater among southwest intervention regions compared to a number of the control regions, but not all. The survey demonstrated the increasing role that social media plays in health communication, which was significantly higher than the state average in the Pilbara, and a tendency toward significance in the Kimberley and Peel regions. There was an inverse trend between use of both social media and the internet (elsewhere) and age, suggesting that these communication methods were more useful at targeting younger age groups. A significantly greater proportion of individuals from the Pilbara and Kimberley (intervention) obtained information regarding mosquitoes and mosquito-borne disease from their workplace, compared to all other regions (Supplementary Table 2).

### Attitudes

Across WA, 22.4% of survey respondents felt mosquitoes posed a health risk where they lived, 39.1% felt they were a nuisance, while 38.5% felt they were of no concern at all (Supplementary Table 3). Respondents from the Kimberley, Pilbara, and Gascoyne regions (intervention) were consistently more concerned about the potential impact of mosquitoes on their health compared to the majority of control groups. A similar regional pattern was noted for the proportion of individuals bitten by mosquitoes on a daily basis. Females were also more concerned about the health risk associated with mosquitoes compared to males and there was a tendency for more females to report being bitten on a daily basis. An occupational risk existed for those individuals working in the Kimberley, Pilbara, Gascoyne, and Goldfields-Esperance regions, while a recreational risk existed for those in the Kimberley and Pilbara (Supplementary Table 3).

### Practices

The most common methods used by respondents to reduce mosquitoes on their property included killing mosquitoes as they noticed them (84.1%) and eliminating stagnant water and/or water holding containers (72.9%) (Supplementary Table 4). The most common personal protection measures to prevent mosquito bites included ensuring mosquito screens were intact (92.1%), applying repellent (75.0%), wearing long-sleeved clothing (67.2%), and staying indoors at peak mosquito times (64.7%). While regional variation existed in the prevention practices adopted, individuals from the Kimberley, Pilbara, and Gascoyne (intervention regions) generally undertook more prevention activities compared to the control groups (Supplementary Table 4). Repellent use was significantly higher in the Kimberley compared to all other regions. Of those individuals who reported using mosquito repellent in the past 12 months throughout the State, the majority had applied a chemical-based formulation (88.6%), with use significantly higher in Leschenault (intervention) than all control regions. The overall finding that 56.0% of respondents reported using a “natural” product, 15.1% reported using repellent wipes/towelettes and 14.2% reported using wearable devices such as repellent bracelets was concerning. There was a tendency for a greater proportion of females to report using natural repellents, compared to males.

### Fight the Bite Evaluation

On average, 8.2% (CI: 4.6–11.9) of survey participants across the state were able to recall *FTB*, of which 4.3% (CI: 1.6–6.9) were unprompted and 3.9% (CI: 1.2–6.6) were prompted responses. A further 13.6% (CI: 10.0–17.3) of individuals recalled other communication material that could not confidently be identified as *FTB* or was unrelated to the campaign. Campaign recollection by survey respondents from the Kimberley and Geographe regions (intervention) was significantly greater than all control groups (Figure 2). Recollection by individuals from the Pilbara and Gascoyne (intervention) was either significantly greater than the control groups or tended toward significance. Recall among those from the Peel and Leschenault regions (intervention) was not significantly different from the controls.

Of the 226 individuals who recalled the campaign, 43.8% reported an increase in awareness of mosquitoes and mosquito-borne diseases (Figure 3). Due to the small number of individuals included in this subset, a regional comparison was not undertaken. A total of 27.4% reported improved prevention behavior and practices, with a further 61.5% reportedly doing everything *FTB* recommends (Figure 4). Key health messages, *Cover Up, Repel*, and *Clean Up*, were recalled by 72.6, 60.2, and 47.8% of the subset of survey participants who recalled the campaign, respectively (Figure 5). *FTB* was most commonly recalled through television, followed by direct local government efforts. A range of other delivery methods were successfully utilized to reach the general public, as shown in Figure 6.

Using the total survey population to estimate the weighted proportion across the state, a total of 1.7% (CI: 0.8–2.6) of individuals in WA experienced an improvement in awareness and 1.2% (CI: 0.3–2.1) changed prevention practices following the introduction of *FTB* (Supplementary Table 2). Awareness was improved in a greater proportion of individuals in the Kimberley (intervention) region (7.0%, CI: 3.6–10.4) compared to four of the control groups and tended toward significance for the remaining two.

## Discussion

The overall campaign recall of 8.2% across WA was considered noteworthy by the authors, given there was no active campaign promotion in control regions and the majority of the Perth metropolitan region, which make up a significant part of the State. Furthermore, the evaluation period was relatively short and minimal paid advertising was undertaken. It is also likely that this figure underestimates the true campaign reach as indirect media exposure generated by *FTB* through newspaper editorials, radio interviews and nightly news coverage resulting from Departmental media statements were classified as *Other Material* (*non*-*FTB*).

It is difficult to draw a comparison between recall rates in this evaluation and other published studies, due to key differences between campaign models. A systematic review of physical activity related campaigns reported recall rates between 17 and 95% (15), whilst sexual health campaigns run within Australia reported rates between 29 and 80% (16). Mass media was widely used as part of the campaign strategies and television advertising was associated with increased reach (15, 16). In contrast, *FTB* relied heavily on community-based initiatives and very little on mass media. Its primary goal was to demonstrate the campaign's ability to raise awareness and improve prevention practices through a sustainable model that leveraged off stakeholder involvement, which has been achieved. The authors acknowledge that in order to maximize reach in future years and have more of an impact on overall awareness and behavior, it will be necessary to invest in a larger media buy (17).

It was pleasing to see that regional differences in recall were evident between the intervention and control groups. This was particularly marked for the Kimberley and Geographe regions, reporting a recall rate of 27.6 and 22.0%, respectively. These rates were significantly higher than all control regions included in the study. Based on interactions with the Department, it was clear that local governments in both regions were highly engaged with *FTB* over the evaluation period. Their efforts consisted of a range of community-based initiatives combined with a modest amount of media advertising. Research demonstrates that this combined approach is the most effective way to generate health behavior change (18). Furthermore, both regions regularly report the highest rate of mosquito-borne disease cases in WA and knowledge of mosquito-borne disease was found to be strong in the baseline study (11). It is therefore likely that residents were more conscious of the disease risk and took more notice of the campaign material. Recall rates in the intervention regions of the Pilbara (17.3%) and Gascoyne (17.6%) were also raised, reflecting proactive campaign promotion once again. Despite remaining comparatively low compared to other published studies, these results demonstrate the potential impact that local governments can have on campaign reach and recall when they are committed to integrating public education into their mosquito management programs.

The proportion of individuals from the Peel region (intervention) who recalled *FTB* was statistically similar to all control groups. This is a surprising finding as active campaign-related community engagement was undertaken and the advertising spend was greater here than in other intervention regions. The apparent lower impact of the campaign is likely attributed to the presence of a more diverse range of competing advertising mediums locally. As Peel is largely an extension of metropolitan Perth, the number of advertising options and the costs associated with these are substantial. With a limited budget, paid advert placement was restricted both in terms of the length of time advertising was sustained for and the chosen medium. Advertising also targeted the City of Mandurah (one of four local governments that make up the Peel region), as this is where the largest number of residents impacted by the region's mosquito issues are located. The responses from individuals within the other local governments in the Peel region, where active engagement and advertising was limited, were included in the evaluation survey for this region which is likely to have had an impact on the results.

Evidence of campaign recall among control group respondents was noted during the evaluation. This observation was likely due to a combination of exposure to *FTB* during travel to intervention regions and exposure to a television advert produced collaboratively by local governments within the Geographe and Leschenault regions. Whilst the advert was due to air in their respective intervention regions only, the Department received reports of it being aired in control regions too as a result of an expanded broadcast area.

Health behavioral changes occur in gradual stages, beginning with an individual first increasing their awareness of a particular issue (3). Whilst only 1.7% of the total survey population reported an increase in awareness due to *FTB*, it is reassuring to see that this was significantly higher in the Kimberley region (7.0%) where recall was greatest. This suggests the awareness change is attributable to campaign exposure. More importantly, exposure to *FTB* increased awareness among almost half of those with campaign recall suggesting that with increased campaign promotion, there is significant potential to have an impact on the general public's knowledge of mosquitoes and mosquito-borne diseases. Key health messages, *Cover Up* (72.6%) and *Repel* (60.2%), were recalled well by those with campaign recall, but further work needs to be undertaken to remind people of the campaign's third call to action, *Clean Up* (47.8%), which involves emptying or covering water-holding containers to prevent mosquito breeding around the home or holiday accommodation. As many individuals in high-risk regions of the State live in close proximity to extensive, natural mosquito breeding habitat, they often report to the Department that they are unable to make an impact on mosquito numbers by reducing breeding on their own property. For this reason, the latter message may not be taken on board and/or recalled as often as the other two personal protection measures.

It was also reassuring that the majority (61.5%) of individuals who recalled the campaign believed they were already doing everything that *FTB* recommended. Notably, campaign exposure improved prevention practices in a further 27.4%. As failure to prompt people to take the necessary steps toward adopting and maintaining a new behavior is often a barrier to the success of health communication strategies, this impact was considered important (3). Snyder and Hamilton's (17) meta-analysis of the effect of health communication on behavior change indicated that on average, campaigns changed 8.0% of the overall population in the positive, expected direction. This was lower for prevention campaigns (3.0%) which are more comparable to *FTB* (17). Whilst the population wide behavioral change for FTB was determined to be 1.2%, there is again significant potential to have an impact on public health with increased campaign reach. In light of these results, the campaign objectives to improve awareness and prevention practices were achieved. Due to the naturally fluctuating nature of mosquito-borne disease incidence in WA, changes in the number of notified cases reported to the Department were not considered to be a valid measure of the campaign's impact.

Whilst it was encouraging to note that only a small proportion of individuals with campaign exposure chose not to change their behavior despite *FTB* recommendations, the survey highlighted the continued use of a range of repellent products that are not proven to be effective (19). A total of 56.0% of individuals who had used a repellent in the past 24 months reported using a natural product containing botanical extracts. Although there is some evidence that selected active botanical extract ingredients will repel biting insects for a short duration (<30 min), they must be reapplied frequently to provide protection comparable to a diethyltoluamide (DEET) or picaridin-based product (20, 21). A further 14.2% of survey respondents reported having used a wearable device, such as a repellent bracelet, despite evidence demonstrating the inefficacy of these products (22, 23). Use of ineffective products was consistently higher in the 35–49 year age bracket. Whilst women were statistically more concerned about the health risk posed by mosquitoes and the likelihood of acquiring a mosquito-borne disease, it was interesting to note the trend that they also chose to use natural repellents more often than men. This may explain the tendency for more women to report being bitten on a daily basis. Research demonstrates it is often more difficult to extinguish an old behavior than it is to adopt a new one (17). In order to effect change, future communication efforts will need to understand and address the reasons why individuals are choosing ineffective products. This will require debunking misconceptions related to the perceived safety of natural repellents and harm associated with more effective chemical-based topical repellents.

The survey indicated that individuals were commonly exposed to general mosquito-related material through similar mechanisms to those noted in the baseline survey (11). While the overall recall of *FTB* messaging on social media was relatively low during the evaluation period (Figure 6), there was an increasing trend for social media use related to general mosquito messaging in the Kimberley, Pilbara and to an extent the Peel, intervention regions between the baseline and the evaluation survey (11) (Supplementary Table 2). Given the potential to reach younger demographic groups who often don't engage with more traditional mediums, this method of communication will become more of a campaign focus into the future.

*FTB* was recalled most commonly through television exposure (Figure 6). On analyzing the open explanations provided in relation to this question, a significant proportion of individuals had seen the advertisement collaboratively produced by local governments that make up the southwest Geographe and Leschenault regions of WA. Given the impact of this advertisement, consideration should be given to increasing the budget for paid television advertising through key regional broadcasting networks in an effort to maximize campaign reach. Unpaid media opportunities created by approaching key current affairs programs with public interest stories on mosquitoes and how to avoid them also proved effective without the requirement for a budget. Local government efforts, including direct mail-outs, active and passive engagement were also highly successful in promoting *FTB*. Radio, displays around town, newspaper advertising and information at medical practices/hospitals were also useful mechanisms, particularly in rural and regional areas where there are a limited number of competing advertising mediums, businesses, and clinics.

There were no obvious significant differences in the general knowledge and practices of individuals between the baseline study undertaken by Potter et al. (11) and that reported here. This is an expected result given the campaign reach wasn't significant enough to have had an impact at this level. The finding that knowledge was greater among individuals living in the intervention regions in the southwest and north of WA, was consistent between both surveys, as was the likelihood for individuals in the north to utilize personal protective measures more often. Unspecified repellent use also across the State remained strong. An occupational risk persists in the Kimberley, Pilbara, Gascoyne, and Goldfields-Esperance regions. The apparent increase in the use of radio and work communication as a means of obtaining mosquito-related information between the two surveys is partially attributed to the addition of the two categories as specific options which were read out to the respondent when answering this question during the evaluation. However, it is also worth noting that multiple local governments, particularly in the Pilbara, encouraged employers in the mining/resources sector to make repellent and *FTB* material available to employees and also advertised at the local airport where fly-in/fly-out (FIFO) workers frequented. These efforts were in response to communication put out by the Department regarding the demonstrated occupational risk highlighted in the baseline survey (11).

Finally, there was a tendency for general attitudes in the present study to rate the impact and biting frequency of mosquitoes as more severe, particularly in the north of the State. Individuals in Kimberley, Pilbara, and Gascoyne (intervention regions) were more likely to view mosquitoes as a health risk in the present study, than as a nuisance. Concern had grown since the baseline survey, where a similar proportion had been split between the two options (11). This likely reflects an increase in the severity of the mosquito problem over the 2016/17 wet season in northern WA, resulting from above average rainfall (December 2016–February 2017) and subsequent mosquito breeding. The number of notified cases of RRV disease then peaked in March 2017, and an outbreak was reported in the west Kimberley at this time (unpublished data, WA Department of Health, 2018). The level of flavivirus activity in sentinel chickens located in the Kimberley and Pilbara also increased compared to previous years (unpublished data, WA Department of Health, 2018).

## Limitations

Funding and timeline constraints were likely to have had a limiting impact on the final reach and evaluation results. It would have also been desirable to determine the nature of the behavior change that resulted from campaign exposure to ensure it was in a positive, predicted direction and did not involve the adoption of competing behaviors that fail to reduce risk (e.g., increased use of an ineffective repellent product). Finally, the number of individuals aged 18–34 years was under represented in this study. This was due to the reduced likelihood that younger individuals used or answered a landline and was difficult to overcome as it was not possible to include mobile phone numbers in the SRC's pool of potential survey participants.

## Conclusion

Health behavior change, in conjunction with effective mosquito control, plays a significant role in reducing the burden of preventable mosquito-borne disease. Whilst local governments in WA have traditionally undertaken effective and responsible control within their own jurisdictions, public education has remained limited. The results of this study demonstrate that *FTB* achieved its objectives in improving awareness and prevention practices among those individuals who were exposed to the campaign. The Department was able to leverage off the reach and involvement of local government within the community, to create a sustainable campaign model that could be introduced to other jurisdictions where local authorities are actively involved in mosquito management.

Despite the promising findings of this evaluation, the overall impact of the campaign across the state remained low. Emphasis now needs to be placed on increasing reach and ensuring the campaign has a sustained positive public health impact into the future (24). Future aims include heightened and sustained promotion of *FTB* by both the Department and local government, as well as expanded collaboration with a range of stakeholders within the community, including Aboriginal environmental health service providers, recreational groups and occupational health and safety advisors in high risk regions who feed into the tourism and mining/resources sectors.

*Fight the Bite* campaign resources can be accessed at: http://ww2.health.wa.gov.au/Articles/F_I/fight-the-bite-campaign.

## Author Contributions

AP developed new and tailored existing FTB resources, project managed the campaign, was primary author of the manuscript, and designed the evaluation survey. AJ assisted in the development of resources, undertook the statistical analysis, reviewed the evaluation survey and manuscript. AM managed the media buy associated with the campaign, and reviewed FTB resources, the evaluation survey and manuscript. ML oversaw the development of FTB resources and campaign budget, and reviewed the evaluation survey and manuscript.

### Conflict of Interest Statement

The authors declare that the research was conducted in the absence of any commercial or financial relationships that could be construed as a potential conflict of interest.
