# Supplementary material for: Evaluation of a Health Communication Campaign to Improve Mosquito Awareness and Prevention Practices in Western Australia
Source: Front Public Health. 2019 Mar 19;7:54. doi: 10.3389/fpubh.2019.00054 (PMC6433780; doi:10.3389/fpubh.2019.00054)
Supplement: Supplementary file 1 [file Table_1.pdf]

Table 1. Demographic breakdown of survey respondents included in the final analysis

Variables	Category	No of Respondents	Proportion within each Category (%)
**Study sites (regions)**	Gascoyne	199	**8.1**
	Geographe	195	**7.9**
	Goldfields-Esperance	199	**8.1**
	Great Southern	197	**8.0**
	Kimberley	198	**8.1**
	Leschenault	194	**7.9**
	Metropolitan Perth	294	**12.0**
	Midwest	195	**7.9**
	Peel	199	**8.1**
	Pilbara	195	**7.9**
	Southwest (other)	195	**7.9**
	Wheatbelt	196	**8.0**
**Gender**	Male	1252	**51.0**
	Female	1204	**49.0**
**Age group (yrs)**	18 – 34	212	**8.6**
	35 – 49	626	**25.5**
	50 – 64	808	**32.9**
	65+	810	**33.0**
**Education**	Primary School	23	**0.9**
	Lower Secondary	398	**16.2**
	Upper Secondary	545	**22.2**
	TAFE/college	642	**26.1**
	University	624	**25.4**
	Postgraduate	164	**6.7**
	Unsure	11	**0.4**
	Refused	49	**2.0**
**Yearly Income ($)**	<50,000	669	**27.2**
	50,000 – <100,000	466	**19.0**
	100,000 – <150,000	358	**14.6**
	150,000 - <200,000	215	**8.8**
	200,000 - <250,000	68	**2.8**
	250,000+	67	**2.7**
	Unsure	129	**5.3**
	Refused	484	**19.7**
