# Supplementary material for: Evaluation of a Health Communication Campaign to Improve Mosquito Awareness and Prevention Practices in Western Australia
Source: Front Public Health. 2019 Mar 19;7:54. doi: 10.3389/fpubh.2019.00054 (PMC6433780; doi:10.3389/fpubh.2019.00054)
Supplement: Supplementary file 2 [file Table_2.pdf]

Table 2. Knowledge of mosquitoes and mosquito-borne disease among respondents, with consideration given to region, gender and age group (with 95% confidence intervals)

Category	Region (%)												Age Group (%)				Gender (%)		State Average (%)
	Kimberley	Pilbara	Gascoyne	Midwest	Goldfields Esperance	Wheatbelt	Perth Metro	Southwest (Peel)	Southwest (Leschenault)	Southwest (Geographe)	Southwest (Other)	Great Southern	18 – 34	35 - 49	50 - 64	65+	Male	Female	
**Correctly able to recognize the following as a locally acquired, mosquito-borne disease in WA**																			
RRV	75.4 (65.4-85.4)	70.9 (62.8-79.0)	**85.0 (75.9-94.0)**	**79.6 (72.9-86.3)**	74.3 (65.8-82.7)	73.5 (66.0-81.1)	58.6 (50.4-66.9)	70.5 (62.0-79.0)	**89.5 (83.7-95.4)**	**78.2 (69.8-86.7)**	**82.0 (75.6-88.3)**	76.5 (67.6-85.4)	49.9 (34.8-65.1)	68.5 (60.1-76.8)	**79.0 (73.4-84.7)**	62.8 (56.2-69.5)	58.4 (50.7-66.2)	69.3 (60.4-78.1)	**63.9 (57.9-69.8)**
BFV	11.2 (7.0-15.5)	5.2 (2.2-8.2)	6.3 (3.1-9.5)	3.2 (0.7-5.8)	3.1 (0.9-5.3)	3.6 (1.0-6.3)	6.3 (0.3-12.3)	4.4 (1.4-7.5)	25.3 (4.0-46.7)	15.2 (10.0-20.4)	9.2 (4.6-13.8)	3.8 (1.3-6.3)	13.7 (1.3-26.0)	2.6 (1.6-3.6)	3.6 (1.7-5.5)	3.7 (1.8-5.5)	2.3 (1.4-3.3)	11.0 (2.8-19.1)	**6.6 (2.4-10.9)**
MVE	**31.9 (23.0-40.8)**	**13.9 (8.7-19.1)**	**17.7 (10.3-25.1)**	9.8 (5.4-14.2)	3.5 (0.6-6.4)	6.6 (3.0-10.2)	2.3 (0.7-3.9)	7.4 (2.7-12.1)	5.1 (1.9-8.4)	7.2 (3.6-10.7)	6.8 (3.6-10.0)	**10.4 (5.9-14.9)**	**1.4 (0.1-2.7)**	5.0 (1.7-8.2)	**5.2 (3.4-7.0)**	**7.6 (4.0-11.1)**	4.4 (2.7-5.6)	4.5 (2.4-6.6)	**4.3 (3.0-5.6)**
WNVKUN	5.1 (0.7-9.5)	3.4 (0.2-6.6)	0.9 (0.0-2.1)	0.5 (0.0-1.6)	-	-	-	-	-	-	-	0.6 (0.0-1.7)	0.2 (0.0-0.5)	0.3 (0.1-0.5)	0.1 (0.0-0.3)	0.0 (0.0-0.1)	0.1 (0.0-0.3)	0.2 (0.1-0.4)	**0.2 (0.1-0.3)**
**What clinical signs are associated with Ross River virus?**																			
Fatigue	72.7 (62.4-83.0)	77.2 (69.8-84.6)	66.6 (56.9-76.3)	79.7 (72.3-87.1)	70.2 (59.2-81.2)	78.2 (68.8-87.6)	67.0 (53.8-80.3)	68.6 (59.7-77.5)	63.8 (54.5-73.1)	69.7 (60.5-79.0)	76.8 (68.8-84.9)	66.4 (56.2-76.7)	69.3 (50.9-87.7)	68.1 (58.4-77.8)	64.2 (56.9-71.5)	63.9 (55.8-71.9)	61.1 (53.0-69.3)	71.1 (62.6-79.7)	**66.6 (60.5-72.6)**
Fever	28.0 (18.4-37.6)	33.8 (24.5-43.0)	28.9 (17.2-40.7)	15.1 (8.6-21.7)	26.2 (17.9-34.4)	27.2 (19.2-35.1)	43.6 (33.5-53.7)	35.0 (26.2-43.7)	21.3 (12.0-30.7)	26.7 (19.0-34.5)	31.9 (22.9-40.9)	35.1 (24.7-45.6)	36.7 (15.4-58.1)	44.2 (34.0-54.4)	33.2 (26.2-40.2)	39.5 (31.2-47.8)	30.5 (23.1-37.9)	45.0 (34.9-55.1)	**38.4 (31.7-45.0)**
Pain/sore muscles	**46.7 (34.5-58.8)**	48.4 (39.3-57.5)	45.7 (35.6-55.8)	46.8 (35.9-57.6)	43.5 (32.5-54.6)	**58.8 (43.9-73.6)**	37.2 (25.3-49.0)	28.9 (20.8-36.9)	35.1 (24.9-45.2)	47.8 (38.2-57.4)	41.8 (32.7-50.9)	43.2 (33.4-53.1)	42.4 (21.3-63.5)	41.9 (21.3-63.5)	34.1 (27.4 -40.9)	27.4 (20.6-34.2)	33.3 (25.9-40.6)	40.7 (30.5-50.9)	**37.3 (30.7-43.9)**
Painful/swollen joints	**66.3 (54.9-77.7)**	46.9 (37.4-56.4)	59.2 (47.2-71.2)	56.1 (44.0-68.2)	**64.7 (54.9-74.5)**	54.1 (44.0-64.2)	42.0 (32.1-51.8)	47.7 (38.1-57.2)	64.9 (51.6-78.2)	59.3 (50.3-68.2)	50.5 (41.2-59.9)	57.2 (46.4-68.0)	41.1 (20.9-61.3)	50.5 (40.4-60.6)	47.2 (40.0-54.5)	47.1 (38.7-55.4)	40.8 (33.1-48.6)	51.4 (41.5-61.4)	**46.6 (40.1-53.1)**
Rash	6.1 (2.4-9.8)	6.9 (1.5-12.3)	6.5 (2.9-10.0)	2.4 (0.0-4.9)	1.9 (0.0-4.0)	6.2 (2.2-10.1)	9.7 (2.4-17.0)	9.7 (1.7-8.6)	23.6 (0.0-47.8)	6.0 (1.4-10.6)	5.4 (2.0 -8.8)	6.3 (2.4 - 10.1)	16.2 (0.0-33.2)	5.7 (1.3-10.2)	7.7 (3.6-11.9)	4.7 (1.5-7.9)	7.1 (1.4-12.8)	10.2 (2.7-17.7)	**8.8 (3.9-13.6)**
**From which sources have you obtained information regarding mosquitoes and mosquito-borne disease?**																			
Health professional	40.5 (30.7-50.4)	31.8 (24.3-39.3)	36.1 (25.6-46.7)	29.0 (20.7-37.4)	30.3 (22.0-38.7)	28.9 (21.3-36.5)	25.6 (18.3-33.0)	21.3 (14.5-28.1)	38.1 (19.8-56.5)	33.3 (24.9-41.7)	26.6 (19.3-33.9)	26.1 (18.7-33.5)	26.4 (12.7-40.1)	32.6 (24.5-40.7)	23.3 (17.8-28.7)	21.2 (15.8-26.5)	23.3 (17.1-29.5)	29.5 (21.2-37.8)	**26.4 (21.2-31.6)**
Friend, family, relative	**64.5 (54.3-74.8)**	64.9 (57.1-72.6)	59.3 (48.3-70.4)	61.8 (52.7-70.8)	**67.6 (59.4-75.9)**	59.9 (51.4-68.4)	50.0 (41.8-58.2)	54.2 (45.7-62.6)	48.8 (33.4-64.1)	47.7 (39.0-56.4)	54.7 (46.4-63.0)	62.9 (53.6-72.2)	60.0 (45.2-74.8)	54.8 (46.1-63.4)	47.4 (40.8-54.0)	40.4 (33.8-46.9)	47.6 (40.1-55.1)	56.9 (48.2-65.5)	**52.2 (46.5-58.0)**
Dept of Health	**51.6 (41.2-62.1)**	**38.1 (30.0-46.3)**	32.7 (22.1-43.3)	19.9 (12.9-27.0)	20.2 (13.4-27.0)	22.8 (16.4-29.3)	22.4 (14.6-30.3)	19.5 (13.4-25.5)	38.5 (20.3-56.7)	28.7 (20.5-36.8)	22.7 (16.3-29.2)	26.0 (17.2-34.8)	28.7 (13.9-43.4)	25.6 (18.2-33.0)	16.2 (12.0-20.5)	20.4 (15.0-25.8)	16.6 (11.8-21.4)	30.5 (21.2-39.7)	**23.5 (18.0-29.0)**
Local Government	**63.0 (52.3-73.7)**	**51.5 (43.1-59.9)**	**53.5 (41.5-65.6)**	27.0 (19.4-34.6)	30.5 (22.7-38.4)	**37.1 (29.4-44.9)**	15.1 (9.8-20.4)	**36.8 (28.6-45.0)**	**46.6 (31.8-61.5)**	**45.2 (36.7-53.7)**	31.6 (23.9-39.4)	22.1 (15.6-28.7)	14.3 (5.2-23.5)	24.6 (17.8-31.3)	22.3 (17.4-27.2)	**32.8 (26.7-38.8)**	20.7 (15.9-25.5)	23.6 (17.3-30.0)	**22.2 (18.2-26.1)**
Social Media	34.4 (24.3-44.6)	**40.8 (32.6-49.1)**	20.5 (9.3-31.7)	18.1 (8.6-27.6)	26.0 (18.4-33.6)	18.0 (10.8-25.1)	21.8 (13.9-29.8)	34.4 (25.9-42.9)	16.9 (8.7-25.1)	12.3 (6.2-18.4)	17.5 (10.1-24.9)	15.1 (6.5-23.7)	38.6 (23.8-53.5)	24.9 (17.7-32.1)	14.2 (9.8-18.5)	**2.8 (1.1-4.6)**	20.6 (13.8-27.5)	25.4 (16.6-34.2)	**23.0 (17.4-28.6)**
Internet (elsewhere)	11.0 (5.0-17.1)	18.6 (12.0-25.1)	**7.4 (3.7-11.1)**	14.2 (2.8-25.7)	11.7 (5.0-18.5)	7.6 (1.9-13.3)	18.6 (11.0-26.2)	18.3 (10.9-25.8)	**6.9 (2.4-11.5)**	14.7 (8.8-20.6)	15.6 (9.9-21.3)	11.3 (3.2-19.3)	28.6 (14.1-43.1)	14.7 (8.8-20.7)	12.8 (8.3-17.4)	**5.4 (2.5-8.4)**	17.4 (11.5-23.3)	17.0 (8.0-25.9)	**17.2 (11.8-22.6)**

Cells shaded green indicate results are significantly higher than the state average.
Cells shaded orange indicate results are significantly lower than the state average.
Region headings shaded grey indicate intervention groups

Table 2. Knowledge of mosquitoes and mosquito-borne disease among respondents, with consideration given to region, gender and age group (with 95% confidence intervals)

Print media	34.2<br>(25.0-43.4)	30.5<br>(22.9-38.1)	21.5<br>(12.0-30.9)	19.6<br>(12.9-26.3)	**18.0<br>(11.6-24.4)**	**18.2<br>(11.4-25.0)**	31.1<br>(23.9-38.3)	31.3<br>(24.0-38.5)	23.3<br>(14.2-32.3)	**48.2<br>(39.5-56.9)**	32.5<br>(24.5-40.5)	23.4<br>(14.9-32.0)		20.2<br>(7.8-32.5)	36.5<br>(28.0-45.0)	33.9<br>(27.6-40.2)	33.9<br>(27.5-40.3)		28.8<br>(22.4-35.2)	31.5<br>(23.6-39.4)		**30.1<br>(25.0-35.2)**
School, college, university	28.9<br>(19.3-38.4)	15.1<br>(9.4-20.9)	15.4<br>(5.1-25.6)	11.9<br>(2.9-20.9)	13.0<br>(6.6-19.5)	10.2<br>(4.0-16.5)	17.2<br>(9.8-24.7)	11.7<br>(5.4-18.0)	10.6<br>(3.7-17.5)	12.1<br>(6.2-18.0)	13.7<br>(8.2-19.1)	**5.1<br>(2.1-8.2)**		25.6<br>(11.3-39.8)	17.4<br>(10.8-24.0)	7.7<br>(4.3-11.0)	**5.4<br>(2.1-8.6)**		13.4<br>(8.1-18.6)	18.0<br>(9.0-27.0)		**15.7<br>(10.4-21.0)**
Television	23.9<br>(15.0-32.8)	25.6<br>(18.5-32.8)	18.2<br>(8.0-28.4)	**19.2<br>(13.2-25.2)**	21.2<br>(14.1-28.2)	21.1<br>(14.0-28.2)	34.5<br>(26.8-42.1)	32.0<br>(24.0-40.1)	27.3<br>(17.1-37.5)	38.2<br>(30.1-46.3)	30.3<br>(22.7-37.9)	**18.3<br>(12.4-24.1)**		28.0<br>(14.0-42.0)	29.0<br>(21.0-37.0)	40.1<br>(33.5-46.7)	34.3<br>(27.7-40.8)		31.4<br>(24.7-38.2)	32.9<br>(24.5-41.3)		**32.2<br>(26.8-37.6)**
Radio	**25.7<br>(16.7-34.8)**	**22.2<br>(15.1-29.3)**	23.5<br>(14.4-32.6)	9.5<br>(5.3-13.6)	13.5<br>(7.9-19.1)	11.5<br>(6.7-16.3)	10.0<br>(6.2-13.8)	17.2<br>(10.5-23.8)	14.9<br>(7.6-22.1)	19.3<br>(13.3-25.3)	17.1<br>(11.4-22.9)	18.8<br>(10.1-27.5)		7.1<br>(1.7-12.4)	14.5<br>(8.5-20.5)	15.6<br>(10.9-20.2)	12.9<br>(8.6-17.2)		14.4<br>(9.9-18.9)	9.7<br>(6.4-12.9)		**12.0<br>(9.2-14.9)**
Work	**44.8<br>(34.4-55.1)**	**46.5<br>(38.1-54.8)**	15.3<br>(9.8-20.8)	10.4<br>(5.6-15.3)	18.8<br>(11.4-26.2)	15.3<br>(9.7-20.9)	13.6<br>(7.5-19.6)	14.1<br>(7.7-20.5)	13.1<br>(7.0-19.3)	9.5<br>(5.0-14.0)	12.5<br>(7.2-17.7)	10.2<br>(2.4-18.0)		15.1<br>(3.9-26.2)	20.7<br>(13.8-27.6)	15.4<br>(11.0-19.7)	**4.1<br>(1.6-6.6)**		15.6<br>(11.0-20.3)	13.8<br>(6.7-20.9)		**14.7<br>(10.5-18.9)**

Cells shaded green indicate results are significantly higher than the state average.
Cells shaded orange indicate results are significantly lower than the state average.
Region headings shaded grey indicate intervention groups
