# Supplementary material for: Evaluation of a Health Communication Campaign to Improve Mosquito Awareness and Prevention Practices in Western Australia
Source: Front Public Health. 2019 Mar 19;7:54. doi: 10.3389/fpubh.2019.00054 (PMC6433780; doi:10.3389/fpubh.2019.00054)
Supplement: Supplementary file 3 [file Table_3.pdf]

Table 3. Attitudes of respondents in regards to mosquitoes and mosquito-borne disease, with consideration given to region, gender and age group (with 95% confidence intervals)

Category	Region (%)												Age Group (%)				Gender (%)		
	Kimberley	Pilbara	Gascoyne	Midwest	Goldfields Esperance	Wheatbelt	Perth Metro	Southwest (Peel)	Southwest (Leschenault)	Southwest (Geographe)	Southwest (Other)	Great Southern	18 - 34	35 - 49	50 - 64	65+	Male	Female	State Average (%)
**What impact do mosquitoes have on your quality of life?**																			
Health risk	**55.2 (44.6-65.7)**	**44.9 (36.5-53.3)**	**46.2 (34.3-58.0)**	20.9 (11.5-30.4)	30.5 (22.3-38.8)	29.2 (20.4-37.9)	19.4 (12.6-26.2)	22.7 (16.1-29.3)	40.8 (23.0-58.6)	31.4 (23.4-39.5)	22.2 (15.2-29.2)	19.2 (10.6-27.7)	23.1 (10.3-35.9)	23.6 (16.6-30.7)	25.5 (19.9-31.2)	15.1 (10.5-19.8)	15.2 (10.7-19.6)	29.6 (21.4-37.9)	**22.4 (17.6-27.2)**
Nuisance	32.6 (22.5-42.6)	33.8 (25.7-41.8)	37.3 (26.1-48.5)	41.5 (30.8-52.3)	41.3 (32.2-50.5)	47.6 (39.0-56.1)	38.8 (31.1-46.6)	38.6 (30.0-47.2)	36.1 (23.8-48.5)	31.8 (23.5-40.1)	44.4 (35.9-52.8)	46.6 (36.7-56.6)	35.8 (22.0-49.7)	44.6 (36.0-53.3)	39.9 (33.4-46.4)	35.4 (28.9-41.9)	44.0 (36.4-51.5)	34.2 (26.7-41.7)	**39.1 (33.6-44.5)**
No concern	**12.3 (4.3-20.3)**	**16.6 (11.0-22.1)**	**21.3 (14.9-27.7)**	**23.3 (16.9-29.6)**	**23.1 (14.0-32.1)**	28.2 (20.0-36.3)	33.4 (25.9-41.0)	34.2 (26.2-42.2)	36.8 (28.4-45.1)	37.6 (28.7-46.4)	38.7 (30.4-47.0)	41.7 (33.5-49.9)	41.1 (25.7-56.4)	31.7 (23.6-39.8)	34.6 (28.2-40.9)	49.5 (42.7-56.2)	40.9 (33.5-48.2)	36.2 (27.1-45.3)	**38.5 (32.7-44.4)**
**During the worst times of the year, how often do you get bitten by mosquitoes?**																			
Everyday	**64.6 (54.7-74.5)**	**41.2 (32.9-49.6)**	**36.0 (25.4-46.6)**	20.0 (12.8-27.3)	**36.2 (27.2-45.2)**	**33.9 (25.5-42.3)**	10.5 (6.0-15.1)	21.0 (13.6-28.4)	18.3 (10.1-26.4)	15.5 (9.3-21.7)	14.2 (8.3-20.1)	12.0 (3.8-20.2)	12.5 (4.4-20.5)	17.6 (11.7-23.4)	20.1 (14.9-25.3)	9.3 (5.4-13.2)	10.7 (7.9-13.5)	19.3 (13.2-25.4)	**15.0 (11.6-18.4)**
**In which locations are you bitten by mosquitoes?**																			
Home	81.5 (73.3-89.7)	68.5 (60.4-76.5)	**89.3 (84.6-93.9)**	77.6 (69.6-85.5)	82.8 (76.8-88.8)	**89.3 (82.8-95.9)**	71.8 (63.0-80.6)	79.8 (72.5-87.1)	**87.2 (81.0-93.4)**	78.3 (70.6-86.0)	80.2 (72.6-87.8)	84.8 (78.1-91.5)	65.3 (49.0-81.7)	76.1 (68.4-83.7)	81.7 (76.3-87.0)	81.3 (75.1-87.6)	76.3 (69.0-83.6)	73.1 (63.0-83.1)	**74.7 (68.5-80.9)**
Recreation	**79.1 (71.4-86.8)**	**71.9 (64.0-79.8)**	58.7 (47.3-70.1)	48.4 (36.1-60.8)	47.1 (37.5-56.8)	37.5 (29.0-46.1)	49.4 (40.7-58.2)	39.2 (30.0-48.3)	41.7 (26.6-56.8)	58.6 (49.3-67.9)	43.6 (34.4-52.8)	48.8 (37.5-60.2)	61.4 (46.5-76.3)	45.3 (36.4-54.2)	46.3 (39.3-53.3)	**32.2 (24.7-39.6)**	47.1 (38.9-55.3)	50.6 (41.6-59.7)	**48.8 (42.7-55.0)**
Work	**50.0 (39.4-60.7)**	**41.7 (33.1-50.3)**	**33.7 (22.7-44.7)**	16.1 (5.5-26.8)	**38.1 (28.4-47.8)**	20.2 (11.6-28.8)	6.7 (1.4-12.0)	13.9 (6.7-21.1)	8.3 (2.3-14.4)	22.9 (14.1-31.8)	15.1 (8.3-21.9)	14.0 (4.5-23.4)	15.7 (5.1-26.3)	12.9 (7.8-17.9)	7.9 (5.2-10.6)	**2.9 (1.7-4.0)**	12.5 (7.9-17.0)	9.8 (3.7-15.9)	**11.1 (7.4-14.9)**
**How much of a problem do you think mosquitoes are where you live?**(1 – not a problem; 4 – significant problem)																			
Rating	**3.3 (3.1-3.5)**	**2.7 (2.6-2.9)**	**2.6 (2.4-2.9)**	2.0 (1.9-2.1)	**2.5 (2.4-2.7)**	**2.5 (2.3-2.6)**	2.0 (1.8-2.1)	2.2 (2.1-2.4)	**2.6 (2.4-2.8)**	**2.3 (2.2-2.5)**	2.0 (1.8-2.1)	2.2 (2.0-2.3)	2.0 (1.7-2.2)	2.3 (2.1-2.5)	2.3 (2.1-2.4)	1.8 (1.7-2.0)	2.0 (1.9-2.1)	2.2 (2.0-2.4)	**2.1 (2.0-2.2)**
**How concerned are you about catching a mosquito-borne disease?** (1 – not concerned; 5 – very concerned)																			
Rating	**3.7 (3.4-4.0)**	**3.3 (3.0-3.5)**	**3.3 (2.9-3.6)**	2.8 (2.5-3.0)	**3.1 (2.8-3.4)**	**3.0 (2.7-3.2)**	2.4 (2.2-2.6)	2.9 (2.6-3.1)	**3.4 (3.1-3.7)**	**3.2 (3.0-3.5)**	2.7 (2.5-3.0)	2.8 (2.5-3.0)	2.4 (2.0-2.8)	2.6 (2.4-2.9)	**2.9 (2.7-3.0)**	2.5 (2.3-2.7)	2.3 (2.2-2.5)	2.8 (2.6-3.0)	**2.6 (2.4-2.7)**

Cells shaded green indicate results are significantly higher than the state average.
Cells shaded orange indicate results are significantly lower than the state average.
Region headings shaded grey indicate intervention groups
