# Supplementary material for: Evaluation of a Health Communication Campaign to Improve Mosquito Awareness and Prevention Practices in Western Australia
Source: Front Public Health. 2019 Mar 19;7:54. doi: 10.3389/fpubh.2019.00054 (PMC6433780; doi:10.3389/fpubh.2019.00054)
Supplement: Supplementary file 4 [file Table_4.pdf]

Table 4. Practices in regards to mosquito avoidance among respondents, with consideration given to region, gender and age group (with 95% confidence intervals)

	Region (%)												Age Group (%)				Gender (%)		
**Category**	**Kimberley**	**Pilbara**	**Gascoyne**	**Midwest**	**Goldfields Esperance**	**Wheatbelt**	**Perth**	**Southwest (Peel)**	**Southwest (Leschenault)**	**Southwest (Geographe)**	**Southwest (Other)**	**Great Southern**	**18 - 34**	**35 - 49**	**50 - 64**	**65+**	**Male**	**Female**	**State Average (%)**
**Which measures do you take to reduce the number of mosquitoes present on your property?**																			
Kill as noticed	91.8 (86.9-96.6)	90.1 (84.6-95.7)	**95.1 (92.2-98.0)**	91.0 (87.0-94.9)	91.0 (85.5-96.5)	90.0 (85.6-94.4)	81.2 (73.8-88.6)	89.0 (83.6-94.4)	94.6 (91.3-97.9)	96.3 (93.8-98.8)	86.4 (81.0-91.9)	90.8 (86.4-95.2)	77.3 (63.0-91.7)	88.6 (82.9-94.3)	89.2 (85.1-93.3)	83.1 (77.9-88.4)	86.4 (81.3-91.5)	81.8 (72.8-90.8)	**84.1 (78.9-89.4)**
Residual spray	26.8 (18.6-35.1)	30.8 (22.8-38.9)	30.4 (18.0-42.8)	32.9 (21.8-43.9)	25.6 (18.1-33.2)	41.4 (32.5-50.4)	31.6 (23.7-39.4)	36.0 (27.6-44.4)	37.3 (18.7-55.9)	25.1 (17.1-33.1)	13.8 (9.0-18.5)	22.2 (15.5-28.9)	39.3 (24.5-54.1)	27.8 (20.2-35.4)	28.3 (22.3-34.3)	27.6 (21.5-33.6)	32.8 (25.3-40.2)	30.5 (22.2-38.8)	**31.6 (26.0-37.2)**
Eliminate stagnant water	**89.8 (83.1-96.5)**	79.4 (71.8-87.0)	**87.2 (80.5-94.0)**	72.4 (61.3-83.5)	79.2 (72.1-86.4)	78.4 (71.3-85.4)	69.3 (61.7-76.9)	79.9 (73.4-86.3)	**88.7 (82.6-94.8)**	80.9 (73.4-88.3)	85.9 (81.0-90.7)	82.8 (74.4-91.2)	66.0 (51.6-80.3)	82.0 (75.1-88.8)	75.3 (69.3-81.3)	68.7 (62.2-75.2)	69.1 (61.5-76.8)	76.7 (69.3-84.1)	**72.9 (67.5-78.3)**
**What measures have you taken in the last 12 months to protect yourself and family from being bitten?**																			
Mosquito coils	**77.2 (67.9-86.6)**	**67.8 (60.1-75.6)**	**72.0 (63.3-80.6)**	47.3 (36.8-57.8)	60.7 (51.5-69.8)	60.4 (52.6-68.3)	44.3 (36.4-52.3)	47.6 (39.0-56.2)	50.1 (34.4-65.8)	54.9 (46.3-63.6)	53.2 (44.9-61.6)	48.1 (38.3-58.0)	39.3 (25.2-53.5)	58.6 (50.1-67.1)	56.6 (50.1-63.2)	32.7 (26.5-39.0)	47.5 (40.0-54.9)	47.1 (38.5-55.8)	**47.3 (41.6-53.0)**
Repellent	**94.0 (91.0-97.0)**	**88.4 (83.3-93.4)**	80.8 (73.3-88.3)	73.0 (65.6-80.4)	79.9 (72.1-87.7)	82.2 (76.6-87.9)	72.5 (64.6-80.4)	79.2 (72.3-86.2)	82.2 (74.9-89.4)	82.4 (76.3-88.5)	77.1 (71.1-83.0)	76.3 (67.7-84.9)	71.7 (56.6-86.7)	82.5 (75.8-89.2)	81.0 (75.8-86.2)	61.7 (55.1-68.3)	78.4 (72.7-84.0)	71.6 (62.3-80.9)	**75.0 (69.4-80.5)**
Clothing	71.2 (61.2-81.1)	**81.6 (75.1-88.2)**	72.1 (61.6-82.5)	66.3 (56.6-76.0)	72.9 (64.4-81.4)	74.9 (68.1-81.7)	65.8 (58.0-73.6)	61.8 (53.4-70.2)	74.8 (65.2-84.5)	75.1 (67.0-83.2)	75.9 (69.5-82.3)	74.6 (66.0-83.3)	68.5 (54.0-82.9)	74.8 (67.2-82.3)	64.0 (57.5-70.5)	57.3 (50.6-64.0)	69.9 (63.4-76.3)	64.5 (55.7-73.2)	**67.2 (61.7-72.6)**
Stay indoors	76.1 (66.8-85.4)	74.1 (66.7-81.6)	72.5 (62.7-82.2)	68.2 (60.2-76.1)	68.0 (59.9-76.2)	72.0 (64.5-79.6)	63.3 (55.6-71.0)	61.3 (52.8-69.8)	77.7 (69.0-86.4)	67.2 (59.1-75.4)	58.7 (50.6-66.9)	73.4 (64.6-82.2)	70.5 (56.5-84.5)	69.4 (61.4-77.4)	62.1 (55.6-68.6)	50.1 (43.3-56.9)	62.4 (55.4-69.4)	66.9 (58.6-75.2)	**64.7 (59.2-70.1)**
Insect screens	**97.8 (95.8-99.7)**	95.0 (91.1-98.8)	91.5 (82.6-100.0)	92.3 (89.0-95.6)	93.0 (88.0-97.9)	96.2 (93.8-98.7)	90.7 (85.6-95.7)	97.1 (94.6-99.7)	95.7 (92.9-98.5)	**97.8 (95.7-99.9)**	90.7 (86.1-95.3)	92.3 (88.0-96.6)	90.2 (80.4-100.0)	96.0 (92.3-99.7)	92.7 (89.3-96.2)	88.9 (84.4-93.4)	97.8 (87.2-96.4)	92.4 (87.1-97.8)	**92.1 (88.6-95.6)**
Operate fans	**72.6 (64.0-81.2)**	44.3 (36.1-52.5)	**62.9 (52.6-73.1)**	37.6 (27.6-47.5)	27.7 (19.6-35.8)	34.9 (26.9-42.9)	34.5 (26.7-42.4)	36.9 (28.8-45.1)	22.2 (13.5-30.8)	33.4 (25.3-41.4)	25.6 (18.7-32.5)	25.2 (17.8-32.7)	32.0 (17.6-46.5)	38.7 (30.2-47.1)	37.0 (30.6-43.4)	31.1 (24.8-37.4)	27.9 (21.6-34.3)	41.6 (32.8-50.4)	**34.8 (29.2-40.3)**
Electronic zapper	18.7 (11.3-26.2)	22.1 (15.1-29.2)	25.6 (13.9-37.4)	19.0 (12.1-25.9)	**37.1 (28.3-45.9)**	28.8 (21.1-36.5)	18.1 (12.6-23.5)	24.6 (17.3-31.9)	21.3 (12.8-29.7)	22.5 (15.6-29.5)	21.4 (14.6-28.3)	17.1 (11.2-23.0)	13.4 (4.9-21.9)	26.0 (18.3-33.6)	22.7 (17.3-28.2)	18.6 (13.6-23.7)	21.2 (15.3-27.2)	18.4 (13.5-23.4)	**19.8 (15.9-23.8)**
Automatic spray	23.5 (16.0-31.0)	42.5 (34.2-50.8)	28.9 (17.3-40.6)	32.2 (22.3-42.2)	36.9 (28.1-45.7)	39.7 (31.4-47.9)	28.8 (21.5-36.1)	36.8 (28.4-45.2)	40.5 (22.6-58.4)	33.1 (24.3-41.8)	22.0 (15.3-28.7)	31.5 (22.3-40.7)	31.2 (17.6-44.8)	32.8 (24.8-40.9)	29.1 (23.2-35.1)	29.1 (22.9-35.4)	27.5 (20.7-34.3)	34.1 (26.1-42.1)	**30.8 (25.6-36.0)**
Mosquito netting	18.7 (9.9-27.5)	**27.4 (19.5-35.2)**	**29.1 (15.9-42.4)**	13.2 (7.3-19.1)	18.2 (11.1-25.4)	13.3 (8.5-18.0)	7.9 (3.5-12.3)	13.4 (7.3-19.5)	13.0 (6.3-19.7)	14.5 (8.2-20.7)	14.5 (8.2-20.9)	10.8 (6.3-15.4)	9.0 (1.3-16.7)	14.3 (8.4-20.2)	10.4 (6.7-14.1)	5.3 (2.8-7.9)	10.2 (7.0-13.5)	10.0 (4.6-15.4)	**10.1 (7.0-13.3)**
**Which mosquito repellent have you used in the past 12 months?**																			
Chemical-based repellent	82.3 (72.9-91.6)	92.4 (88.0-96.8)	92.0 (87.6-96.4)	89.1 (82.6-95.6)	87.4 (82.2-92.7)	88.7 (81.6-95.7)	88.0 (81.2-94.8)	89.9 (84.6-95.2)	**97.9 (95.8-100.0)**	92.2 (88.1-96.2)	89.3 (83.1-95.5)	81.8 (74.7-88.9)	84.9 (71.8-98.0)	90.7 (85.2-96.2)	90.9 (87.1-94.8)	87.9 (82.2-93.7)	88.9 (82.9-94.9)	88.1 (81.0-95.3)	**88.6 (83.9-93.2)**
Repellent wipes/towelettes	18.9 (9.1-28.7)	11.9 (6.6-17.3)	16.6 (3.0-30.1)	**6.1 (2.1-10.2)**	**6.2 (2.7-9.7)**	17.5 (9.8-25.3)	16.7 (10.2-23.3)	13.5 (6.7-20.3)	9.6 (4.1-15.0)	13.1 (6.4-19.7)	**6.7 (2.8-10.6)**	7.0 (3.1-10.9)	13.9 (2.8-25.0)	19.5 (11.5-27.6)	12.0 (7.4-16.7)	13.9 (7.7-20.1)	15.9 (8.8-22.9)	14.2 (9.0-19.5)	**15.1 (10.6-19.6)**
Wearable devices (eg. bracelet)	10.9 (3.1-18.7)	16.3 (10.4-22.2)	21.6 (6.7-36.5)	9.4 (3.7-15.0)	13.1 (7.0 19.2)	15.5 (9.4-21.5)	14.6 (8.5-20.7)	8.6 (3.7-13.5)	28.1 (3.1-53.1)	14.0 (7.5-20.5)	9.7 (4.0-15.4)	14.7 (4.3-25.1)	11.7 (1.9-21.5)	26.3 (17.6-35.0)	8.7 (4.8-12.6)	4.3 (1.2-7.4)	13.9 (7.5-20.3)	14.5 (9.0-20.0)	**14.2 (9.9-18.4)**
Any natural-based repellent	66.7 (56.3-77.0)	62.9 (54.0-71.8)	60.4 (51.8-76.2)	56.7 (43.6-69.8)	68.0 (59.3-76.7)	64.9 (55.8-74.0)	52.8 (43.6-62.0)	64.6 (55.1-74.0)	48.9 (30.5-67.3)	69.0 (59.2-78.8)	62.0 (52.0-71.9)	60.2 (50.1-70.3)	48.5 (31.9-65.1)	65.0 (56.0-73.9)	61.2 (53.9-68.4)	45.3 (36.9-53.7)	49.0 (40.3-57.7)	63.7 (54.7-72.8)	**56.0 (49.7-62.4)**

Cells shaded green indicate results are significantly higher than the state average.
Cells shaded orange indicate results are significantly lower than the state average.
Region headings shaded grey indicate intervention groups
